# Predicting Outpatient Follow‐Up Retention After Inpatient Treatment in Patients With Alcohol Use Disorder: A Data‐Driven Random Forest Approach

**DOI:** 10.1111/adb.70169

**Published:** 2026-06-12

**Authors:** Jennifer J. Barb, Lillian C. King, Alexandria N. Hughes, Jenny Yarmovsky, Nancy Diazgranados, Mehdi Farokhnia, Gwenyth R. Wallen, Melanie Schwandt, Lorenzo Leggio

**Affiliations:** ^1^ Translational Biobehavioral and Health Promotion Branch, Clinical Center National Institutes of Health Bethesda Maryland USA; ^2^ Division of Intramural Clinical and Biological Research National Institute on Alcohol Abuse and Alcoholism Bethesda Maryland USA; ^3^ Clinical Psychoneuroendocrinology and Neuropsychopharmacology Section, Translational Addiction Medicine Branch, National Institute on Drug Abuse Intramural Research Program and National Institute on Alcohol Abuse and Alcoholism Division of Intramural Clinical and Biological Research National Institutes of Health Baltimore Maryland USA; ^4^ Center for Alcohol and Addiction Studies, Department of Behavioral and Social Sciences Brown University Providence Rhode Island USA; ^5^ Division of Addiction Medicine, Department of Medicine, School of Medicine Johns Hopkins University Baltimore Maryland USA; ^6^ Department of Neuroscience Georgetown University Medical Center Washington DC USA

**Keywords:** alcohol use disorder, machine learning, outpatient AUD treatment, random forest

## Abstract

Longer treatment engagement is associated with improved recovery outcomes in alcohol use disorder (AUD), making patient retention a critical determinant of reduced return to drinking. This study aimed to identify factors predicting outpatient treatment engagement, operationalized as the number of follow‐up visits, among individuals with AUD following inpatient care. We applied a five‐step analytic framework integrating random forest modelling (RFM) and Least Absolute Shrinkage and Selection Operator (LASSO) regression to identify predictors of outpatient visit frequency. Clinical, psychological and physiological variables (*n* = 177 per participant) collected during inpatient treatment prior to discharge were included. RFM ranked variables associated with follow‐up visits, with LASSO used for validation and complementary selection. Over a 5‐year period, 119 treatment‐seeking patients with AUD (mean age = 45.8) returned for outpatient care, averaging 5.14 visits, following a medical treatment inpatient stay. Positive urgency (VIP = 44.31) and positive life events (VIP = 41.19) emerged as the strongest predictors; both inversely associated with visit frequency. LASSO confirmed positive urgency as a significant predictor (coefficient: −0.03296). Greater alcohol use severity and higher haemoglobin levels were also associated with fewer outpatient visits, whereas higher depressive symptom severity predicted increased follow‐up engagement. Using complementary machine learning and regression approaches, this study identified affective traits, alcohol use severity and physiological factors as key determinants of outpatient engagement following inpatient AUD treatment. Interestingly, positive urgency and positive life events, often considered markers of recovery or resilience, were linked to reduced outpatient attendance, suggesting that certain personality or motivational factors may diminish perceived need for continued care. These findings highlight the importance of integrating psychological and motivational variables into postdischarge planning to enhance retention and improve early recovery outcomes.

## Graphical abstract

Among 119 treatment‐seeking individuals with Alcohol Use Disorder (AUD), machine learning and regression analyses identified psychological and physiological factors predicting outpatient treatment engagement following inpatient care. Positive urgency and positive life events were the strongest predictors and were associated with fewer follow‐up visits. Greater alcohol use severity and hemoglobin levels also predicted reduced engagement, whereas depressive symptom severity predicted increased attendance. These findings highlight key affective and biological determinants of treatment retention after discharge from inpatient AUD treatment.

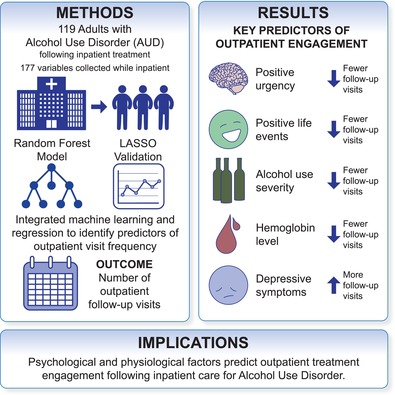

**Abbreviations**
AUD: alcohol use disorder
ICD: International Classification of Diseases
LASSO: Least Absolute Shrinkage and Selection Operator
NIAAA: National Institute on Alcohol Abuse and Alcoholism
NIH: National Institutes of Health
RFM: random forest model
RMSE: root mean squared error

## 1 Introduction

Alcohol use disorder (AUD) is a chronic and relapsing medical condition characterized by persistent and harmful alcohol consumption that leads to significant morbidity and mortality [1, 2]. In 2024, approximately 27.9 million Americans aged 12 years and older were estimated to have AUD. Despite its high prevalence, fewer than one in four individuals with AUD ever seek or receive treatment [3, 4]. A population‐based US study reported that only 8% of people meeting AUD criteria had received any form of treatment in the past year [3].

Treatment‐seeking people with AUD consistently exhibit greater alcohol severity than those who do not seek treatment, whereas comparative studies have shown that treatment seekers typically have higher AUD severity, greater drinking intensity and more DSM‐defined symptoms than non–treatment‐seeking individuals [5, 6, 7]. Machine learning classifiers have reliably distinguished treatment seekers from nonseeking people based on markers of severity, including drinking behaviour, AUD symptoms and depressive features [5]. While severity markers distinguish individuals who initiate treatment, considerably less is known about factors that influence continued engagement once care has begun. Given that sustained outpatient participation is critical in a chronic, relapsing disorder like AUD, understanding predictors of follow‐up attendance represents an important next step.

Because of the chronic and cyclical nature of AUD, a return to drinking following treatment is common. Most individuals experience at least one drinking event after inpatient or outpatient treatment [6, 7]. A 1‐year study found that participants frequently transitioned between remission and relapse, and only 25% maintained continuous remission throughout the year [8]. Similarly, readmission to treatment programmes is common; one study reported a 22% readmission rate during the 10‐year observation period [9]. Encouragingly, longer treatment duration has consistently been associated with improved recovery outcomes and lower rates of readmission [9].

Given the strong link between treatment duration and recovery success, identifying factors that promote retention in AUD treatment is of clinical importance. However, predictors of continued outpatient engagement are likely multidimensional, reflecting interactions among psychiatric symptoms, alcohol‐related severity, physiological measures and demographic characteristics. Traditional analytic approaches, such as regression‐based models, typically rely on linear and additive assumptions and require prespecification of interaction terms, which may limit their ability to capture complex, non‐linear relationships among interdependent variables. In addition, these approaches may be less effective in the presence of correlated clinical features and are often optimized for inference on individual predictors rather than overall predictive performance. As a result, they may not fully capture the multivariate patterns that influence treatment engagement. In contrast, data‐driven predictive modelling approaches can accommodate high‐dimensional data, model non‐linear relationships and identify interactions without requiring a priori specification, providing a complementary framework for understanding factors associated with outpatient return visits.

This study assesses data collected between 2015 and 2019 from the National Institute on Alcohol Abuse and Alcoholism (NIAAA) Natural History Protocol. The sample population included in this work was treatment‐seeking individuals with AUD who elected to participate in the outpatient component of the programme within 4 weeks of completing an approximate 4–6‐week inpatient programme. Using a data‐driven approach, this study aimed to identify psychological, physiological, alcohol‐related and demographic factors that predicted the number of outpatient visit returns to the National Institutes of Health Clinical Center (NIH CC) following discharge from the inpatient stay among newly abstinent individuals with AUD. Understanding these predictors may help optimize patient retention strategies, improve long‐term treatment outcomes and reduce rates of relapse.

## 2 Methods

### 2.1 Study Population

All participants were treatment‐seeking patients with AUD and were enrolled in the NIAAA Natural History Protocol (14‐AA‐0181, NCT02231840) inpatient treatment programme at the NIH Clinical Center. Diagnosis was determined according to DSM‐IV criteria for Alcohol Dependence and/or Alcohol Abuse or DSM‐5 criteria for AUD [10, 11]. The study was approved by the appropriate NIH Institutional Review Board. All participants provided written informed consent. The participants completed a standardized battery of clinical assessments and self‐reported questionnaires during inpatient treatment. Included participants completed up to approximately 4–6 weeks of inpatient treatment at the NIH CC and following discharge, elected to return for outpatient treatment. Participants were invited to return up to 12 times, once per week, over the course of 16 weeks. On occasion, some participants were allowed to return more than 12 times for a total of a maximum of 16 visits. Outpatient treatment included group therapy, a shared meal with other participants and the opportunity to continue to fill prescriptions and to discuss health‐related concerns with their clinical care team at NIH. Any reference in the current work to relapse indicates a return to drinking.

### 2.2 Overview of Included Variables

A total of 29 psychological assessments, 11 demographics variables and 73 physical health markers already collected were used in this data‐driven approach for prediction. For a full description of the methods, see Supporting Information. The full data matrix before cleaning included 403 variables per participant, which included subcomponents contributing to an overall total score. Subscores or subcomponents of scores were not included in the final data matrix. Self‐reported residential zip codes were used to calculate the distance (in miles) between their homes and to the NIH CC (zip code: 20892), using the R package ‘zipcodeR’ [12, 13]. A summary table and abbreviations of the measures included is outlined in Table S1. For a description of all clinical assessments and variables, including full details for alcohol‐related measures, psychological and physiological assessments, see Supporting Information. To identify comorbid conditions, clinicians conducted a clinical evaluation during the history and physical examination and documented any relevant diagnoses in the NIH CC electronic medical records. Each participant's chart was reviewed, and any relevant comorbid conditions were assigned diagnostic codes based on the International Classification of Diseases (ICD‐9 for those enrolled prior to September 2015 and ICD‐10 for those enrolled thereafter) [14, 15].

The Charlson Comorbidity Index (CCI) was calculated to quantify the number of diagnosed comorbidities based on ICD codes [16].

### 2.3 Data Analysis and Statistical Procedure

**FIGURE 1:**
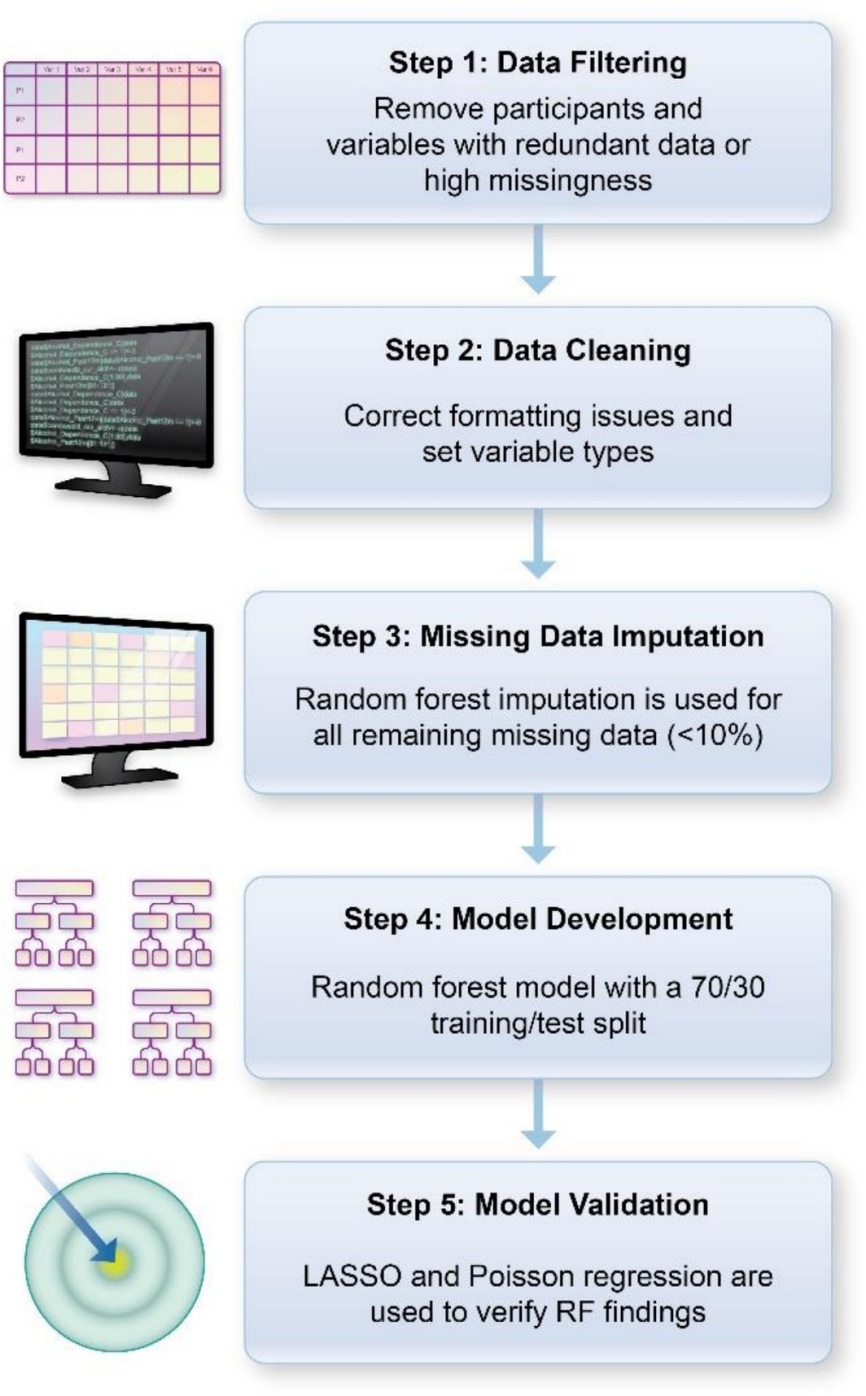
Analysis procedure workflow. Data analysis flow. Step 1, data filtering: Variables with redundant or high missingness are removed. Step 2, data cleaning: corrects formatting issues in the data. Step 3, missing data imputation: Random forest (RF) is used to impute the remaining missing data to preserve the largest possible dataset. Step 4, model development: RFM is created. Step 5, model validation: The LASSO and Poisson regression are used to check the validity of the RF findings.

JMP statistical discovery software (SAS Headquarters, Cary, NC) and R Studio (CRAN Project) were used for data analysis, cleaning and visualization. The CCI was computed using the *Comorbidity* R package, and the resulting scores were included as an input variable in the RFM [17]. Pearson correlation was used to assess the association between calculated distance and the number of follow‐up visits for each participant. A five‐step data analysis procedure was employed (Figure 1); a detailed description of each step is included in the Supporting Information. Briefly, the five steps included the following: (1) data filtering to remove participants and variables with excessive missingness or redundancy, resulting in a final dataset of 119 participants and 177 variables per participant; (2) data cleaning to correct formatting issues, recoding of categorical variables and removing irreconcilable measures such as race; (3) data imputation using random forest–based imputation (R package *missForest*) for variables with < 10% missing data [18]; (4) model development using an RFM (R package *randomForest*) with a 70/30 train‐test split, 1000 trees and RMSE for performance evaluation [19]; and (5) model validation through Least Absolute Shrinkage and Selection Operator (LASSO) Poisson regression (R package *glmnet*) and follow‐up Poisson modelling to assess the directionality and statistical significance of top predictors [20].

## 3 Results

### 3.1 Participants

Patient demographics and clinical characteristics are summarized in Table 1. Participants were seeking treatment and predominantly middle‐aged (mean = 45.8 years) adults with educational attainment approximating a high school level or higher and body mass indices in the overweight range. The majority of the participants were single (58%), and one individual did not specify marital status. The cohort was largely male (63%), racially diverse and more commonly single (58%). Most individuals entered inpatient treatment without formally documented medical comorbidities, although a subset reported one or more co‐occurring conditions. Prior to admission, participants demonstrated long‐standing and severe alcohol use, reflected by chronic heavy drinking patterns and elevated alcohol use severity indices. At baseline, clinically meaningful symptoms of anxiety and depression were observed. No clinically significant elevations were observed in markers of hepatocellular injury (ALT and AST), cholestasis (ALP and GGT) or bilirubin levels (see Table 1 and the Supporting Information).

**TABLE 1:** Study population characteristics.

Variable	Mean (SD) or *N* (%)
	(*N* = 119)
**Age (years), mean (SD)**	45.8 (11.6)
**BMI (kg/m** ^**2**^ **), mean (SD)**	27.17 (4.82)
**Sex, *N* (%)**	
Male	76 (63.8%)
**Race, *N* (%)**	
White	62 (52%)
Black	47 (39.5%)
Other (Multiracial, Asian)	6 (5.0%)
Unknown	< 5 (< 4.2%)
**Years of education, mean (SD)**	13.7 (3.0)
**Marital status, *N* (%)**	
Divorced/separated/widowed	30 (25.2%)
Married	19 (16%)
Single	69 (58.0%)
**Household income, *N* (%)**	
< $10k	31 (26.1%)
$10k–$29.9k	28 (23.5%)
$30k–$49.9k	27 (22.7%)
$50k–$74.9k	9 (7.6%)
> $75k	24 (20.2%)
**Distance from the NIH Clinical Center (miles), *N* (%)**	20.82 (37.8)
**Number of comorbid conditions, *N* (%)**	
0	86 (72.3%)
1	30 (25.2%)
≥ 2	3 (2.5%)
**Alcohol use related measures, mean (SD)**	
AUDIT	28.3 (6.0)
ADS	21.5 (8.5)
CIWA‐Ar (peak)	4.7 (3.1)
OCDS	19.2 (10.0)
Average of total drinks over 90 days	11.4 (8.1)
Heavy drinking days (over 90 days)	66.1 (27.8)
Heavy drinking years	14.8 (10.1)
**Psychological assessment measures, mean (SD)**	
Baseline BSA	12.4 (6.5)
Baseline MADRS	16.8 (8.9)
BSA^a^	4.9 (4.2)
MADRS^a^	5.6 (5.3)
**Number of outpatient visits, mean (SD)**	5.14 (3.8)
**Liver biomarkers (baseline), mean (SD)**	
Total bilirubin (mg/dL)	0.7 (1.0)
ALT (U/L)	46.4 (50.3)
AST (U/L)	61.6 (70.6)
ALP (U/L)	85.8 (62.5)
GGT (U/L)	171.9 (370.3)

Abbreviations: ADS = Alcohol Dependance Scale; ALP = alkaline phosphatase, ALT = alanine aminotransferase; AST = aspartate aminotransferase; AUDIT = Alcohol Use Disorder Identification Test; BMI = body mass index; BSA = Brief Scale for Anxiety; CIWA‐Ar = Clinical Institute Withdrawal Assessment for Alcohol‐Revised; GGT = gamma‐glutamyl transferase; MADRS = Montgomery–Åsberg Depression Rating Scale; NIH = National Institutes of Health; OCDS = Obsessive Compulsive Drinking Scale.

^a^ Assessed at inpatient Day 23.

### 3.2 Variables Predictive of Number of Follow‐Up Visits

An RFM was trained on 177 demographic, psychological, clinical and alcohol‐related variables to identify predictors of outpatient follow‐up visits. Household income, a categorical variable containing levels for different income ranges, stood out as the most informative predictor of follow‐up visits, so additional models were fit with income coded numerically (Figure S1). Model performance, evaluated by root mean square error (RMSE), showed minimal change between the least and most predictive models (ΔRMSE = 0.08; Table S2), indicating limited influence of the coding of the income variable on predictive accuracy. The final RFM achieved an RMSE of 3.64. Variable importance (VIP) scores for all predictors are provided in Table S3, with the top 90th percentile VIPs (18 variables) displayed in Figure 3 and Table 2. The three most influential predictors from the RFM were positive urgency (VIP = 44.31), defined as the tendency to act impulsively in response to positive emotional states; positive events score (VIP = 41.19), reflecting the frequency or impact of recent positive life events; and average drinks per day (total drinks over the last 90 days) (VIP = 31.0) (Table 2).

**TABLE 2:** Top random forest predictors with directionality and significance.

		RFM output		Poisson regression output	
Rank	Predictor	VIP score	Direction (effect on # visits)	Poisson *p*‐value	Significant?
1	Positive urgency	44.31	Down	0.11	No
2	Positive life events	41.20	Down	**< 0.0001**	Yes
3	Avg. drinks per day	31.03	Down	**0.009**	Yes
4	Free triiodothyronine (T3)^c^	30.74	Down	0.60	No
5	Total drinks	28.47	Down	**0.009**	Yes
6	Hemoglobin^c^	23.62	Down	**0.009**	Yes
7	Triglycerides^c^	22.79	Up	**0.03**	Yes
8	CRP^c^	19.23	Down	0.07	No
9	Haemoglobin^b^	19.13	Down	**0.009**	Yes
10	Vitamin B12^c^	18.50	Up	0.65	No
11	ADS score	18.38	Down	**0.0008**	Yes
12	Extraversion factor (NEO)	17.83	Down	0.34	No
13	Heavy drinking days	17.25	Down	0.37	No
14	TSH^c^	16.85	Up	0.79	No
15	Diastolic BP	15.93	Up	0.68	No
16	Haematocrit^c^	15.46	Up	0.12	No
17	Depression^d^	14.87	Up	—	—
18	Depression^a^	14.27	Up	**0.0002**	Yes

*Note:* Ranked by VIP score from RF model. Direction of effect and significance output from *p*‐value based on Poisson regression model. Direction of ‘Down’ indicates a negative association with the number of follow‐up visits, and ‘Up’ indicates a positive association with the number of follow‐up visits. Significance and bold: *p* < 0.05. Average drinks per day is a proxy for total drinks because the variables are colinear.

Abbreviations: ADS, alcohol dependence scale; CRP, C‐reactive protein; D1, Day 1; D2, Day 2; D9, Day 9; NEO, NEO Five‐Factor Inventory; TSH, thyroid stimulating hormone.

^a^ Time of collection: baseline.

^b^ Time of collection: Day 1.

^c^ Time of collection: Day 2.

^d^ Time of collection: Day 9.

### 3.3 Assessment of Directionality on Return Visit Prediction

**FIGURE 2:**
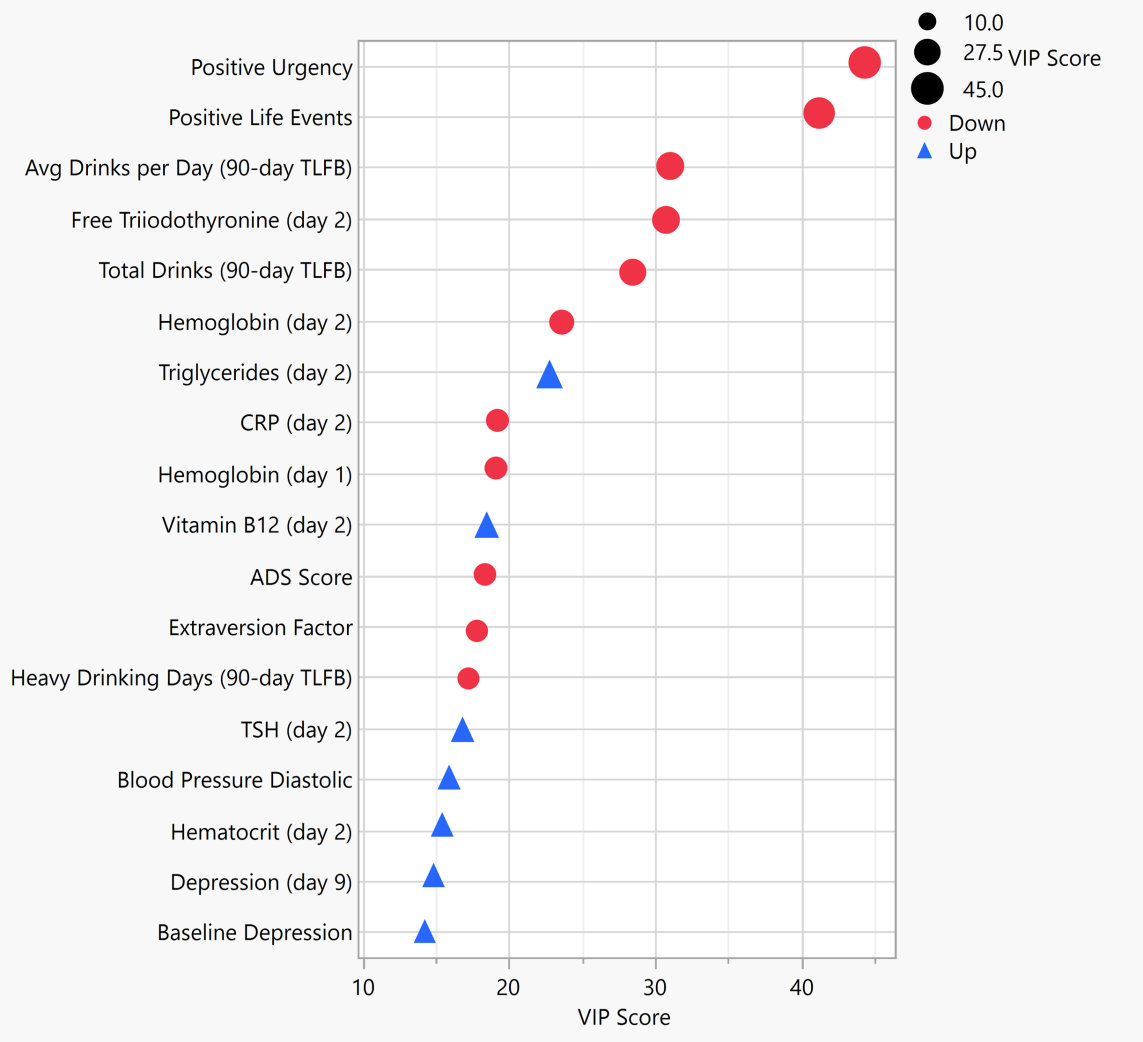
Variable importance predictors and directional association plot for the 90th percentile variables. Top 90th percentile variables (18 out of 177) based on the RFM. Size of the variable indicates VIP score size, and colour and marker (red circle = Down; blue triangle = Up) indicate direction of influence (using LASSO) on outpatient follow‐up visits based on Poisson regression analysis.

To assess the directionality of associations between key predictors and outpatient follow‐up visits, a Poisson regression model was employed using the top 90th percentile of variables identified by the RFM (Figure 2). Eight of the 18 variables were significantly associated with the number of follow‐up visits (Table 2 and S4). Notably, the top VIP variable identified by the RFM, positive urgency score, was not significantly associated with the outcome using Poisson regression (*p* = 0.11). Greater alcohol severity and dependence, as indicated by total drinks (*p* = 0.009) and ADS score (*p* < 0.001), were associated with fewer follow‐up visits. Among physiological biomarkers, two variables were significant predictors of follow‐up visits. Elevated baseline triglycerides were associated with more frequent follow‐up visits (*p* = 0.03), while greater baseline haemoglobin levels (at Days 1 and 2) were associated with fewer follow‐up visits (*p* = 0.009). Among the psychological measures, greater baseline depressive symptom severity (assessed by MADRS) (*p* < 0.001) was associated with more frequent follow‐up visits while a higher positive life events score (*p* < 0.001) was associated with fewer follow‐up visits.

### 3.4 Lasso Regression as a Complement for Important Variable Selection

To complement the RFM analysis, we applied a LASSO Poisson regression model as an alternative method for variable selection. Whereas the RFM identified a broader set of potentially important predictors, the LASSO model yielded a more conservative subset, selecting only three variables from the full set of 177. Because LASSO imposes strong penalization, it functions as a high‐threshold filter that retains only predictors with the most robust linear associations with the outcome.

The LASSO‐selected predictors were ADS score (coefficient: −0.00059), positive urgency (coefficient: −0.03296) and baseline creatinine (coefficient: 0.31190). Of the three selected predictors, ADS score and positive urgency overlapped with variables identified in the top 90th percentile of RFM importance scores (Figure 2), suggesting consistency across methods for these features. In contrast, baseline creatinine emerged uniquely from the LASSO model, indicating that this physiological measure may have a direct association with outpatient follow‐up visits; this marker was not captured by the RFM.

### 3.5 Association of Follow‐Up Visits With Distance to Outpatient Treatment Site

**FIGURE 3:**
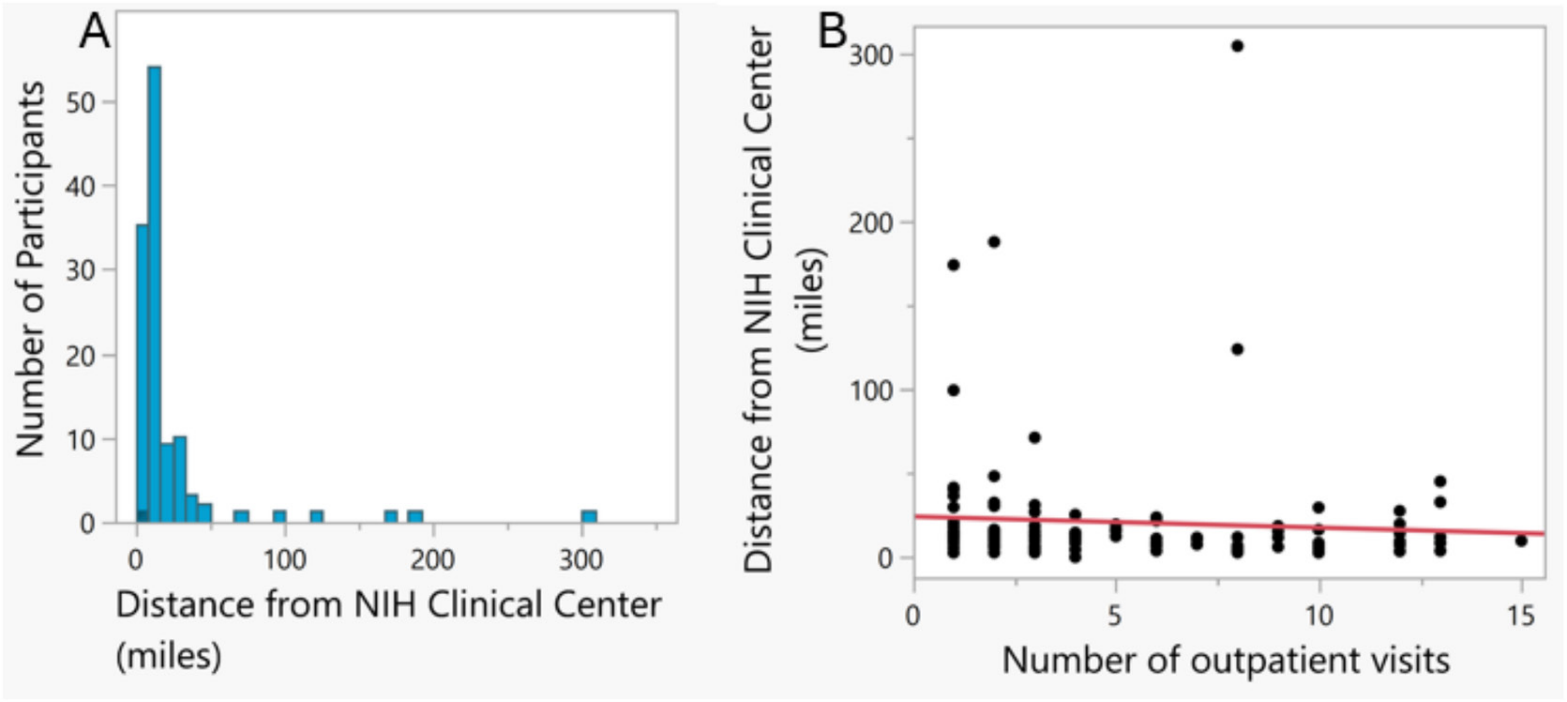
Association between distance from outpatient treatment and participant reported zip code residence. (A) Distance to the NIH Clinical Center based on participant zip code (x‐axis) and the count of participants binned by distance in miles (y‐axis). (B) Correlation assessing distance from patient reported residence zip code to NIH Clinical Center outpatient treatment programme to the number of return outpatient visits. Pearson correlation coefficient: −0.066 and *p* = 0.473.

Outpatient treatment sessions were scheduled once per week for up to 16 weeks at the NIH CC with a maximum of 15 follow‐up visits. On average, participants returned to the programme about five times (mean = 5.14, SD = 3.8), ranging from 1 to 15 visits, with six patients returning more than 12 times (Table 1, Figure 3A). To evaluate whether distance from participant‐reported residential zip codes to the NIH CC zip code was associated with the number of follow‐up visits, the distance between the two sites was calculated. Participants lived an average of 20.82 miles away. No significant association was observed between distance and number of follow‐up visits (*p* = 0.47; Figure 3B).

## 4 Discussion

In this study, we applied a data‐driven, multivariate framework to identify variables associated with outpatient follow‐up visits following inpatient treatment among patients with AUD. A major advantage of this type of analysis was the deep phenotyping of the participants who underwent standardized, comprehensive assessments while in the alcohol treatment inpatient unit at the NIH CC. While the NIAAA Natural History protocol collected a wide range of variables, the current analysis focused on 177 demographic, psychological, physiological and alcohol‐related measures, providing a unique opportunity to evaluate which patient characteristics were associated with return for outpatient follow‐up care. Analyses of this type may be extended to broader clinical settings to generate data‐informed hypotheses regarding treatment response, patient trajectories and potential targets for personalized interventions.

Our analytic strategy integrated machine learning via a RFM for prediction with LASSO Poisson regression to estimate the direction and statistical significance of associations with outpatient visit frequency. These complementary methods converged on a set of predictors capturing both alcohol‐related severity and psychological functioning, revealing several consistent patterns. Notably, distance from the NIH Clinical Center was not significantly associated with the number of return visits in regression analyses and did not emerge as a predictor in any of the applied models.

The RFM identified multiple influential predictors (top 90th percentile VIP scores), notably positive urgency, positive life events and average drinks per day (as the top 3 VIPs). Positive urgency, a dimension of impulsivity reflecting a tendency to engage in rash or poorly controlled behaviour during states of elevated positive affect [21], has been increasingly implicated in alcohol use, AUD and relapse vulnerability. For example, we previously showed that negative urgency and positive urgency contribute to the relationship between adult attention‐deficit/hyperactivity disorder symptoms and AUD severity in patients with AUD [22]. However, the role of urgency in AUD remains largely underexplored in relation to treatment engagement. In the present study, higher positive urgency was associated with fewer outpatient follow‐up visits, suggesting that individuals prone to impulsive behaviour during positive emotional states may be less likely to maintain structured treatment engagement. One possible explanation is that during periods of elevated mood or perceived well‐being, individuals high in positive urgency may deprioritize treatment engagement and adherence, acting on immediate positive reinforcement rather than long‐term recovery goals. This pattern may reflect a reduced perceived need for continued care, therefore disrupting follow‐up attendance.

Similarly, positive life events [23], often conceptualized as indicators of improving psychosocial context or recovery capital, may influence motivation for continued care by altering perceived need for structured treatment or support. Previous research has indicated that both affective traits and alcohol‐related factors contribute to treatment engagement [24, 25, 26].

Consistent with prior evidence, patients with more severe AUD are at greater risk for early relapse and possible reduced treatment adherence [5, 27]. In the present study, Poisson regression identified a subset of clinical and physiological variables associated with outpatient follow‐up frequency. Specifically, higher alcohol severity (total drinks on the TLFB and higher ADS score) was associated with fewer follow‐up visits, whereas greater depressive severity (MADRS score) predicted more frequent follow‐up visits. Among physiological biomarkers, elevated baseline triglycerides were associated with higher outpatient engagement, while elevated haemoglobin was associated with fewer returns. Chronic heavy alcohol use has been associated with haematologic abnormalities, including anaemia and macrocytosis, through mechanisms such as nutritional deficiencies, bone marrow suppression and alcohol‐related liver dysfunction [28, 29, 30]. Heavy alcohol use has also been linked to dyslipidemia, including elevated triglycerides [31]. These biomarkers may reflect alcohol‐related physiological effects; however, the nature and directionality of their relationships with treatment engagement remain unclear and warrant further investigation.

Interestingly, the top VIP from the RFM, positive urgency, did not reach statistical significance in the Poisson model (*p* = 0.11), suggesting that while it may contribute to overall variance, its direct effect on visit frequency is less robust. This pattern may indicate that individuals experiencing heightened positive affect or improved life circumstances perceive less need for structured treatment support, which may contribute to reduced follow‐up attendance, whereas those with greater depressive symptoms may rely more on outpatient care as a coping mechanism or source of social support. The lack of statistical significance in the Poisson model (*p* = 0.11) may reflect differences in model assumptions, as the RFM captures non‐linear relationships and interactions that are not explicitly modelled in regression‐based approaches. Collectively, these findings point to a complex interplay among alcohol severity, affective symptoms, physiological health and treatment engagement during the post‐inpatient recovery phase of AUD, as identified through a data‐driven analytic approach.

Recovery from AUD is increasingly understood as a dynamic process of behaviour changes following a trajectory towards improvements in wellness, rather than strictly abstinence or symptom reduction [32]. Contemporary recovery models emphasize heterogeneity in goals, timing and support needs, with individuals moving between periods of stability, vulnerability and re‐engagement with care. From this perspective, reduced outpatient attendance may not uniformly reflect disengagement or poor outcomes, but may instead reflect shifting recovery needs, increased perceived self‐efficacy or reliance on alternative sources of recovery capital. Alternatively, sustained engagement with outpatient services may represent adaptive help‐seeking during periods of heightened emotional distress or risk. Together, our findings and the conclusions here discussed are consistent with the recent NIAAA research definition of recovery [33] as they reflect the importance of conceiving recovery as both a process of behavioural change and an outcome as well as the importance of taking into account not only abstinence sensu stricto but also features related to biopsychosocial functioning and quality of life.

The complementary use of LASSO regression supported these findings, selecting only ADS score and positive urgency among the three selected variables. Additionally, LASSO also selected creatinine, which was not selected by the RFM as one of the strongest predictors, further emphasizing the modest predictive power of many other variables. The difference in selected predictors between the two models likely reflects the modest predictive performance of the RFM, raising the possibility that some RF‐identified predictors may be less stable or more sensitive to noise. The LASSO results therefore provided a complementary perspective on key variables associated with outpatient return behaviour. While machine learning approaches like random forest models are useful for ranking VIP, regression‐based methods provide clarity on directionality and statistical significance, and combining these two approaches helped to gain clarity on how the variables were associated with the number of follow‐up visits.

Overall, these results suggest that for this cohort of 119 treatment‐seeking patients with AUD who elected to return for outpatient treatment after discharge from an inpatient treatment programme, psychological traits, affective states and alcohol severity may be more salient determinants of outpatient treatment engagement than demographics, biomarkers, comorbid conditions or other factors. Specifically, positive urgency and severity of AUD emerged as the strongest determinants of future outpatient treatment engagement following an inpatient treatment programme.

### 4.1 Limitations

Several limitations of this study should be acknowledged. First, while the collection of relapse‐related information was attempted during the outpatient treatment period, these data were significantly limited due to the majority of patients ultimately being lost to follow‐up. This limited our ability to directly link treatment engagement to post‐inpatient alcohol use outcomes. Second, psychological and physiological assessments were conducted only at the start and throughout the inpatient programmme, but not repeatedly during the outpatient treatment window, which restricted our ability to capture dynamic changes in affective or cognitive states during newly abstinent individuals, which also may have influenced retention outcomes. Additionally, participants were enrolled in a single treatment programme within a specific research‐oriented clinical setting (the NIH CC), which may limit the generalizability of these findings to other settings. Furthermore, the sample size of 119 individuals was relatively small. This work included a comorbidity index calculation based on ICD codes. Given that ICD diagnosis only counts certain comorbidities, the final number assessed for the current work is not comprehensive and could be misrepresented from the true number of comorbidities for this patient cohort. In addition, data on participants' engagement in mutual aid groups (e.g., Alcoholics Anonymous and SMART Recovery) or other recovery support services (e.g., peer recovery coaching) during the outpatient follow‐up period were not routinely or consistently collected. As such, although the importance of these programmes in AUD recovery is established [34, 35], we were unable to account for the potential influence of external recovery supports on treatment engagement and visit frequency. Other potentially relevant factors that were not assessed include employment status, housing stability and caregiving responsibilities, which may also have influenced outpatient follow‐up. Future studies should incorporate longitudinal, time‐aligned measurements of psychological, behavioural and biological variables to more accurately identify factors that influence outpatient treatment engagement and relapse risk.

### 4.2 Potential Clinical Implications and Interpretation

These findings have important potential implications for optimizing post‐inpatient care and tailoring outpatient interventions for patients with AUD. The association between greater alcohol severity and lower treatment engagement highlights the need for enhanced transitional support for those exiting inpatient programmes with higher severity of AUD and/or recent heavy drinking patterns. Prior studies have shown that people with greater AUD severity are more likely to relapse within the first month postdischarge [7, 24], emphasizing that this window represents a critical period for close monitoring and targeted relapse prevention strategies. Incorporating early monitoring, motivational enhancement, contingency management and/or other retention strategies immediately following discharge may help sustain engagement among these high‐risk individuals.

The differential associations observed for depressive symptoms and positive affect‐related variables suggest that emotional and motivational factors also shape treatment adherence. Increased depressive symptoms predicting greater outpatient attendance may reflect an adaptive reliance on structured emotional support through group therapy during periods of low mood. These quantitative findings support previous qualitative evidence in this population describing the need to sustain sober social support networks postdischarge to prevent relapse [36]. Conversely, the finding that higher positive life events and positive urgency were linked to fewer follow‐up visits may indicate that people experiencing elevated positive affect or impulsivity are less likely to perceive ongoing treatment and long‐term care as necessary once immediate distress subsides. This pattern aligns with prior work indicating that positive emotional impulsivity is associated with risk‐taking and poor adherence across populations with neuropsychiatric disorders [25, 26, 37]. Addressing such affect‐driven fluctuations in motivation, through adaptive scheduling, personalized feedback or brief digital interventions, could improve retention for those prone to disengagement when mood or external circumstances improve.

Together, these results demonstrate that outpatient retention among people with AUD can be influenced by both clinical severity and affective regulation processes, suggesting that prediction models incorporating psychological variables may better identify people at risk for dropout than demographic or logistical factors alone. While distance from the treatment site was not related to the number of follow‐up visits in this sample, emotional and behavioural predictors emerged as more salient determinants of engagement, highlighting the need for multidimensional models of aftercare planning that integrate clinical, psychological and contextual data.

## Author Contributions

J.J.B., L.C.K., and L.L. conceived and designed the study. J.J.B., L.C.K., and A.N.H. drafted the initial manuscript. L.C.K. and A.N.H. conducted the statistical analyses. J.J.B., L.C.K., and A.N.H. developed the figures and data visualizations. M.S. curated, cleaned, and provided the dataset for analysis. N.D. oversaw the patient population and primary clinical protocol. J.Y. assisted with manuscript formatting, editing for clarity, and preparation of the final submission. G.R.W. and L.L. acquired funding for the study. M.F. contributed to study interpretation and manuscript development. All authors reviewed, edited, and approved the final manuscript.

## Funding

This study was supported by (1) National Institutes of Health (NIH) intramural funding ZIA‐DA000635 and ZIA‐AA000218 (Clinical Psychoneuroendocrinology and Neuropsychopharmacology Section), jointly supported by the Intramural Research Program (IRP) of the National Institute on Drug Abuse (NIDA) and the Division of Intramural Clinical and Biological Research (DICBR) of the National Institute on Alcohol Abuse and Alcoholism (NIAAA); (2) the NIAAA DICBR Office of the Clinical Director (OCD); and (3) the NIH Clinical Center.

## Conflicts of Interest

Outside his NIH work, Dr. Leggio receives an honorarium from UK Medical Council on Alcohol as Editor‐in‐Chief for Alcohol and Alcoholism, and he also receives royalties from Rutledge as co‐editor of a textbook.

## Supporting information


**Table S1:** Assessments included in random forest and abbreviation of measure.
**Table S2:** Out‐of‐sample test error for random forest models with different household income (HHI) variables.
**Table S3:** See additional spreadsheet of random forest model results: [Supplemental Table S3.xls].
**Table S4:** Poisson regression model for number of visits using the top 90th percentile RF #3 predictors.
**Figure S1:** Random forest variable importance scores for the top 90th percentile with three different household income variables.

**Data S1:** Supporting information.

## Data Availability

Data that support the findings in this manuscript are not available to protect patient privacy. Code for all data processing and model fitting is available on GitHub at https://github.com/LCKing02/RFretention.
