# Supplementary material for: Predicting Outpatient Follow‐Up Retention After Inpatient Treatment in Patients With Alcohol Use Disorder: A Data‐Driven Random Forest Approach
Source: Addict Biol. 2026 Jun 12;31(6):e70169. doi: 10.1111/adb.70169 (PMC13263546; doi:10.1111/adb.70169)
Supplement: Supplementary file 1 — Table S1: Assessments included in random forest and abbreviation of measure. Table S2: Out‐of‐sample test error for random forest models with different household income (HHI) variables. Table S3: See additional spreadsheet of random forest model results: [Supplemental Table S3.xls]. Table S4: Poisson regression model for number of visits using the top 90th percentile RF #3 predictors. Figure S1: Random forest variable importance scores for the top 90th percentile with three different household income variables. [file ADB-31-e70169-s001.docx]

**Supplemental Material**

**Supplemental Methods**

**Demographic variables**

Demographic variables were collected at the time of participant consent to the National Institute on Alcohol Abuse and Alcoholism Natural History Protocol. These variables included age, sex, race, years of education, marital status, and household income. Race was self-reported at study enrollment and categorized as White, Black, Multiracial, Asian, or Unknown. To maximize the protection of participant confidentiality, categories with small cell counts (<5) were combined into an “Other” category and/or masked in Table 1.

**Alcohol and nicotine use measures**

To comprehensively characterize alcohol use history, a series of clinical and alcohol-related measures were administered at the start of inpatient treatment. These assessments provided detailed information on drinking behaviors, Alcohol Use Disorder (AUD) severity, and related symptomatology. The 90-day Timeline Followback (TLFB) and lifetime drinking history (LDH) measures were used to assess recent and lifetime drinking behaviors.^1, 2^ The Penn Alcohol Craving Scale (PACS) was used to assess participants alcohol craving over the past week, and the Clinical Institute Withdrawal Assessment for Alcohol - Revised (CIWA-Ar) was used to assess current degree and severity of alcohol withdrawal.^3, 4^ The CIWA-Ar was administered at intake and on the first three days of inpatient treatment (plus as needed, if medically indicated) and the PACS was administered on the 5^th^, 12^th^, and 19^th^ days of treatment. The Alcohol Use Disorders Identification Test (AUDIT), Addiction Severity Index (ASI), Alcohol Dependence Scale (ADS) and Obsessive Compulsive Drinking Scale (OCDS) were each given once at the beginning of participants in-patient stay, and were used to assess overall AUD-related symptomology and phenotypic characteristics.^5-8^ Finally, the diagnosis of AUD was confirmed using the Structured Clinical Interview for DSM IV or DSM 5 Disorders.^9, 10^ The Fagerstrom Test for Nicotine Dependence was used to assess the intensity of physical addiction to nicotine.^11^

**Psychological health measures**

As part of the clinical assessment for the inpatient treatment program, participants completed a battery of standardized instruments measuring various aspects of psychological health (**Supplemental Table S1**). Most assessments were completed around the 8^th^ day of inpatient treatment, to avoid potential confounding related to acute alcohol withdrawal. The Comprehensive Psychopathological Rating Scale (CPRS)^12^, which comprises the Brief Scale for Anxiety (BSA) and the Montgomery–Åsberg Depression Rating Scale (MADRS) was given on intake, day 9, 16, and 23 during treatment. The Wechsler Abbreviated Scale of Intelligence - 2 Scale (WASI-2 scale)^13^ and the NEO Personality Inventory- Revised (NEO)^14^, were administered on day 21 of treatment. These assessments aimed to capture a comprehensive profile of participants' mental and emotional functioning. To evaluate broad personality traits and underlying psychological factors, participants completed the Childhood Trauma Questionnaire (CTQ) to assess experiences of childhood trauma, the Early Life Stress Questionnaire (ELSQ) for early life stress, and the World Health Organization Quality of Life assessment (WHOQOL) to gauge overall life satisfaction.^15-17^ Personality traits were assessed using the NEO, which measures the five major dimensions of personality.^14^ Intellectual functioning was estimated with the WASI-2 scale.^13^ The Columbia Suicide Severity Rating Scale (CSSRS) was used to assess suicidality, and the Perceived Stress Scale (PSS) was used to assess stress.^18, 19^ Participants were also assessed for Anorexia Nervosa and Bulimia Nervosa using the Structured Clinical Interview for DSM IV or 5 Disorders (SCID-IV and SCID-5, respectively), and were assessed for ADHD symptoms using the Adult ADHD Symptom Rating Scale (ASRS).^9, 10, 20^

Sleep quality was assessed using the Pittsburg Sleep Quality Index (PSQI).^21^ The UPPS-P Impulsive Behavior Scale, Revised (UPPS) including the Positive Urgency Scale and the Barratt Impulsiveness Scale (BIS) were used to assess participants impulsivity, and the Buss Perry Aggression Questionnaire (BPAQ) was used to assess aggression.^22-24^ The Life Events Questionnaire (LEQ) was used to determine the number of major life events a participant had experienced in the past year, and the Yale Food Addiction Scale (YFAS) was used to assess food addiction in participants.^25^

**Physiological health variables**Blood samples for assessment of general physical well-being and potential biomarkers were collected using no more than 55 ml of whole blood. The physiological health variables collected included standard liver biomarker assessments, lipid panels, and nutritional status variables. All physiological variables collected were performed using blood tests, urinalysis, and vital signs. All data were collected by clinical and research staff at the National Institutes of Health (NIH) while participants were inpatient at the NIH Clinical Center. See **Supplemental Table S3** for the full list.

**Steps for Analysis workflow**

***Step 1****: Data filtering to remove redundancy and missing*
The original dataset included 131 participants and 403 variables encompassing demographics, clinical surveys, physiological and biomarker data, and psychological assessments. Twelve participants were excluded due to a mostly incomplete data set, resulting in a final sample of 119 participants. Likewise, 226 of the 403 variables were removed due to redundancy or high levels of missingness, leaving 177 variables in the final dataset. A summary table of all included variables is outlined in **Supplemental Table S1**. After data filtering, the final data matrix consisted of 21,063 (119x177) cells in total, with less than 1% of missing data across the entire data matrix before data imputation.

***Step 2****: Data cleaning to remove character symbols and set variable types*
Some physiological variables had limited instances of “<” signs indicating that a patient’s value was below a specified lower limit; these characters were dropped (e.g., <0.2 became 0.2). The presence of non-numeric symbols or words in a numeric variable, with the absence of any numeric value, was set to be missing. Variables with values that were coded as “negative” or “positive” were recoded to 0 and 1, respectively. Multiple estimated Glomerular Filtration Rate **(**eGFR) variables were coded depending on race (an approach now longer used, see Delgado et al., 2022)^26^, causing each variable to have extensive missingness. Due to this irreconcilable missingness, the eGFR variables were excluded from our models.
Categorical variables were explicitly converted to factor variable types. Household income was originally coded as categorical on a 1-9 scale. In the primary Random Forest (RF), this variable was treated as categorical because the spacing between the levels was uneven. We fit two additional random forests (2) including and (3) replaced by the variable recoded with the midpoints of the income ranges represented by the 1-9 values.

***Step 3****: Data Imputation for Missing Data*
To handle missing data (~1%) random forest imputation was used to predict the missing values. The package ‘missForest’ was used to do the missing data imputation in R (CRAN project).^27^ The decision was made to impute missing data for variables missing less than 10% of their data due to concerns that excluding participants or variables due to small amounts of missingness would drastically reduce sample size, available variables, and power of the model.

***Step 4****: Random Forest Model Implementation*
Seventy percent of the data were randomly selected for training the random forest model, and the remaining 30% was retained as a test set used at the end to test the random forest accuracy by Root Mean Squared Error (RMSE). Following the test/train split, data in each set were centered and scaled. The final model was selected after adjustments were made to the household income variable, and models using the original variable were compared to models using the adjusted variable. This process and the other models are shown in the supplemental document as **Supplemental Figure S1** and **Supplemental Table S2**. To evaluate the model performance of this RF, the model was used to predict the number of visits from the 30% of the data reserved to compose the holdout test set. The difference between the predicted and known number of visits were used to compute the out-of-sample RMSE. The R package *randomForest* was used due to its ability to handle numeric and categorical predictors.^28^ The outcome was treated as numeric. Random forests were fit with 1000 trees using 1/3 of the predictors sampled as candidates at each split. The RMSE was calculated for the test set using the differences between the predicted and actual values.

***Step 5****: LASSO and Poisson Regression as validation and insight into predictor influence on retention*
Variables with an RF importance score above the 90^th^ percentile were entered into a Poisson regression model to determine the directionality of the relationship between each predictor and the outcome. The R package stats “*glm*” function with family = “*poisson*” was used to fit the model. Significance was set at the alpha = 0.05 level.

As a complementary approach to identify the variables that were most influential on the number of return visits, we used LASSO (least absolute shrinkage and selection operator) Poisson regression. LASSO regression shrinks most variable coefficients to zero, selecting the most important variables to remain in the model; we address the distribution of the number of return visits by modeling them as a count (Poisson). LASSO depends on a regularization parameter lambda, which we determined using 10-fold cross-validation to select the value of lambda that had minimum mean cross-validated error. LASSO was used in conjunction with the random forest model as a secondary method for identifying important predictors. The R package *glmnet* was used for fitting LASSO Poisson regression models.^29^ 10-fold cross validation was used to select the lambda parameter that minimized mean cross-validation error. All resulting variables with non-zero coefficient estimates were reported.

**Random forest model for predicting the number of outpatient return visits**
A random forest model was fit to the complete set of training data (177 variables) to identify the most impactful variables on the outcome (number of return visits to the outpatient treatment program). The top 90^th^ percentile most important variables are shown in **Supplemental** **Figure S1A**. Household income, treated as a categorical variable with 9 income ranges, stood out as the most important variable. To further investigate the role of household income in predicting return visits, a continuous version of the variable was created containing the midpoints of each income range (i.e., $30,000 - $39,999 changed to $35,000and so on). After the creation of this second household income variable, two further models were fit: one with the new version as a categorical income variable in addition to (**Supplemental** **Figure S1B**), and another with categorical income replaced by (**Supplemental** **Figure S1C**) the continuous household income variable. **Supplemental** **Figure S1B** shows the top 90^th^ percentile of variables when both the original and the new versions of the household income variables were used, whereas **Supplemental** **Figure S1C** shows the top 90^th^ percentile variables when only the new version of household income was used.

To compare model performance for these three random forests, we used each of the three models to predict the number of visits from the 30% of the data reserved to compose the holdout test set. The difference between the predicted and known number of visits were used to compute the out-of-sample root mean square error (RMSE). When the RMSE values of the three models are compared, the model containing only the numeric income variable shown in **Supplemental** **Figure S1C** performed best, with an RMSE of 3.64 (**Supplemental** **Table S2**). A list of all variables included as categorical is shown in **Supplemental Table** S**3.**

**Supplemental Results**

**Liver biomarkers test results**Liver biomarkers assessed and reported here were performed on days 1 and/or days 2 during the inpatient treatment. Liver biomarkers included: alanine aminotransferase (ALT), aspartate aminotransferase (AST), alkaline phosphatase (ALP), gamma-glutamyl transferase (GGT), and total bilirubin. In this patient cohort of treatment seeking individuals with AUD, several liver biomarkers were elevated relative to typical clinical reference ranges, as expected from a clinical standpoint (**Table 1**). ALT was near the upper limit of normal (mean = 46.1 U/L, SD = 50.3). AST was elevated (mean = 61.6 U/L, SD = 70.6), exceeding the typical upper limit (~40 U/L) and consistent with a pattern commonly seen in alcohol-associated liver disease, where AST levels often exceed ALT.^30^ ALP remained within normal limits (mean = 85.8 U/L, SD = 62.5; normal range ~44–147 U/L), while GGT was substantially elevated (mean = 171.9 U/L, SD = 370.3L), well above the upper limit (~60 U/L). Total bilirubin on Day 1 was within the normal range (mean = 0.7 mg/Dl, SD = 1.0). CRP was slightly elevated (mean = 5.4 mg/dL, SD = 16.4; normal <1 mg/dL).

**Supplemental Tables**

**Supplemental Table S1: Assessments included in Random Forest and Abbreviation of Measure**

| **Measure Name and abbreviation** | **Citation** | **Brief description** | **Time Points** | **Broad Category** |
| --- | --- | --- | --- | --- |
| Pittsburg sleep quality index (PSQI)^21^ | (Buysse et al., 1989) | The PSQI assesses subjective sleep quality via questionnaire. | Day 2 | Psychological |
| Timeline Followback (TLFB)^2^ | (Sobell & Sobell, 1992) | The TLFB assesses daily drinking behaviors over the past 90 days. | ~Day 8 | Alcohol |
| Lifetime Drinking History (LDH)^31^ | (Skinner & Sheu, 1982) | The LDH assesses alcohol use retrospectively throughout their lifespan. | ~Day 8 | Alcohol |
| Penn Alcohol Craving Scale (PACS)^3^ | (Flannery et al., 1999) | The PACS assesses tonic or stable experiences of alcohol craving. | Day 5, 12, and 19 | Alcohol |
| Clinical Institute Withdrawal Assessment for Alcohol – Revised (CIWA-Ar)^4^ | (Sullivan et al., 1989) | The CIWA-Ar assesses physical symptoms of alcohol withdrawal and guides the need for medications to treat withdrawal symptoms. | Intake, Day 1, 2, and 3 | Alcohol |
| Comprehensive Psychopathological Rating Scale (CPRS)^12^ | (Åsberg et al., 1978) | The CPRS is comprised of two assessments, the MADRS (Montgomery–Åsberg Depression Rating Scale) and the BSA (brief scale for anxiety), which assess depression and anxiety respectively | Intake, Day 9, 16, 23 | Psychological |
| Structured Clinical Interview for DSM IV Disorders (SCID IV)^9^ | (First, 1997) | The SCID IV is an assessment given by a qualified trained professional assessing for a wide range of DSM diagnoses. In this study participants were assessed for eating disorders using the SCID IV. | ~Day 9 | Psychological |
| Structured Clinical Interview for DSM 5 Disorders (SCID 5)^10^ | (First et al., 2016) | The SCID 5 is an assessment given by a qualified trained professional assessing for a wide range of DSM diagnoses. In this study participants were assessed for eating disorders using the SCID 5. | ~Day 7 | Psychological |
| Alcohol Dependence Score (ADS)^7^ | (Skinner & Allen, 1982) | The ADS is a questionnaire that assesses alcohol dependence. | ~Day 8 | Alcohol |
| Alcohol Use Disorders Identification Test (AUDIT)^5^ | (Saunders et al., 1993) | The AUDIT is a questionnaire that screens for hazardous alcohol use in participants. | ~Day 8 | Alcohol |
| Obsessive Compulsive Drinking Scale (OCDS)^8^ | (Anton, 2000) | The OCDS is a questionnaire that assess thoughts and behaviors around alcohol for obsession and compulsions with alcohol. | ~Day 8 | Alcohol |
| Smoking History Questionnaire (SMHQ)^32^ | (Strong et al., 2014) | The SMHQ assesses smoking behaviors over the lifetime. | ~Day 8 | Substance |
| UPPS-P Impulsive Behavior Scale – Revised (UPPS)^23^ | (Lynam et al., 2007) | The UPPS-P assesses impulsive behavior in the realms of Urgency,  Premeditation, Perseverance, Sensation Seeking, and Positive Urgency. | ~Day 8 | Psychological |
| Buss Perry Aggression Questionnaire (BPAQ)^22^ | (Buss & Perry, 1992) | The BPAQ evaluates aggressive behavior. | ~Day 8 | Psychological |
| Columbia Suicide Severity Rating Scale - Lifetime/Baseline Version (CSSRS)^18^ | (Posner et al., 2011) | The CSSRS is a clinician administered assessment that evaluates the severity and intensity of participants suicidality currently and over their lifespan. | ~Day 1 | Psychological |
| Childhood Trauma Questionnaire (CTQ)^15^ | (Bernstein et al., 1998) | The CTQ assesses traumatic experiences of participants during their childhood and adolescent hood. | ~Day 8 | Psychological |
| Early Life Stress Questionnaire (ELSQ)^16^ | (McFarlane et al., 2005) | The ELSQ assesses exposure to specific stressors in their early life. | ~Day 8 | Psychological |
| Perceived Stress Scale (PSS)^19^ | (Cohen et al., 1983) | The PSS assesses how often and to what degree participants evaluate situations in their lives as stressful, and their ability to cope. | ~Day 8 | Psychological |
| State-Trait Anxiety Inventory For Adults (Form Y-2) (STAIT)^33^ | (Spielberger, 1983) | The STAI is an assessment comprised of two scales, one assessing current (state) anxiety levels, and the other assessing general (trait) anxiety levels in participants. | ~Day 8 | Psychological |
| World Health Organization Quality of Life Assessment (QOL)^17^ | (WHOQOL, 1998) | The QOL is a generic multidimensional assessment covering a broad spectrum of factors influencing the quality of life. | ~Day 8 | Psychological |
| Wechsler Abbreviated Scale of Intelligence - 2 Scale (WASI)^13^ | (Wechsler, 1999) | The WASI is an intelligence test used in adults. | ~Day 21 | Psychological |
| Adult ADHD Self-Report Scale (ASRS)^20^ | (Kessler et al., 2005) | The ASRS is a short self-report symptom checklist designed to quickly assess participants for symptoms of ADHD. | ~Day 8 | Psychological |
| NEO Personality Inventory- Revised (NEO)^14^ | (Costa & McCrae, 1992) | The NEO is a personality assessment designed to evaluate participants personality within the five-factor model. | ~Day 21 | Psychological |
| Yale Food Addiction Scale (YFAS)^25^ | (Gearhardt et al., 2009) | The YFAS evaluates symptoms of food addiction. | ~Day 8 | Psychological |
| Life Events Questionnaire (LEQ)^34^ | (Norbeck, 1984) | The LEQ assesses major life events they have experienced in the past year, marking them as positive or negative. | ~Day 8 | Psychological |

Footnote: Timepoint is based on an up to 28-day inpatient stay. ‘~’ indicates median of the time frame used to collect multiple assessments.

**Supplemental Table S2. Out-of-sample test error for random forest models with different household income (HHI) variables**

|  | **Random Forest Model** | **Out of sample RMSE** |
| --- | --- | --- |
| Model 1 | HHI as categorical | **3.72** |
| Model 2 | HHI as categorical, with an additional numeric HHI variable containing income midpoints | **3.69** |
| Model 3 | HHI as numeric income midpoints only | **3.64** |

**Supplemental Table S3: See additional spreadsheet of random forest model results: [Supplemental Table S3.xls]**

**Supplemental Table S4. Poisson regression model for number of visits using the top 90^th^ percentile RF #3 predictors**

| **Term** | **Estimate** | **Exponentiated Estimate** | **Estimate standard error** | **statistic** | **p-value** |
| --- | --- | --- | --- | --- | --- |
| (Intercept) | 2.43 | 11.4 | 0.658 | 3.7 | 0 |
| Triglycerides^c^ | 0.001 | 1 | 0.001 | 2.15 | **0.032** |
| Total Drinks (TLFB)^d^ | 0 | 1 | 0 | -2.61 | **0.009** |
| Hemoglobin^b^ | -0.186 | 0.83 | 0.072 | -2.6 | **0.009** |
| CPRS^a^ Depression | 0.02 | 1.02 | 0.005 | 3.73 | **0** |
| CPRS^d^ Depression | -0.012 | 0.988 | 0.008 | -1.51 | 0.13 |
| ADS^d^ | -0.019 | 0.981 | 0.006 | -3.35 | **0.001** |
| Positive Life Events | -0.014 | 0.987 | 0.003 | -3.9 | **<0.0001** |
| Positive Urgency^d^ | -0.106 | 0.9 | 0.066 | -1.6 | 0.109 |
| Extroversion Factor | -0.004 | 0.996 | 0.004 | -0.955 | 0.34 |
| Diastolic BP | 0.002 | 1 | 0.004 | 0.415 | 0.678 |
| Hematocrit^c^ | 0.06 | 1.06 | 0.038 | 1.57 | 0.117 |
| Hemoglobin^c^ | 0.028 | 1.03 | 0.13 | 0.218 | 0.827 |
| CRP_D2^c^ | -0.009 | 0.991 | 0.005 | -1.8 | 0.072 |
| Heavy Drinking Days (TLFB) ^d^ | -0.002 | 0.998 | 0.002 | -0.905 | 0.366 |
| Vitamin B12^c^ | 0 | 1 | 0 | 0.461 | 0.645 |
| TSH^c^ | 0.009 | 1.01 | 0.032 | 0.272 | 0.785 |
| Free Triiodothyronine (T3)^c^ | -0.053 | 0.948 | 0.1 | -0.53 | 0.596 |

Footnote: Abbreviation: BP: blood pressure; ADS: Alcohol Dependance Scale; CRP: C-reactive protein; CRPS: Comprehensive Psychopathological Rating Scale; TLFB: 90-day Timeline Followback;; TSH: Thyroid-Stimulating Hormone; T3: triiodothyronine; Time of collection: ^a^Baseline, ^b^Day 1, ^c^Day 2, ^d^Day 9; bold indicates p<.05.

**Supplemental Figures**

**Supplemental Figure S1:** Random Forest variable importance scores for the top 90th percentile with three different household income variables

**Figure SEQ Figure \* ARABIC 1. Variable importance plots for the top 90^th^ percentile important variables in three random forest models.** (A) Household income (HHI) as categorical, (B) HHI as categorical, with an additional numeric HHI variable containing income midpoints, and (C) HHI as numeric income midpoints only.


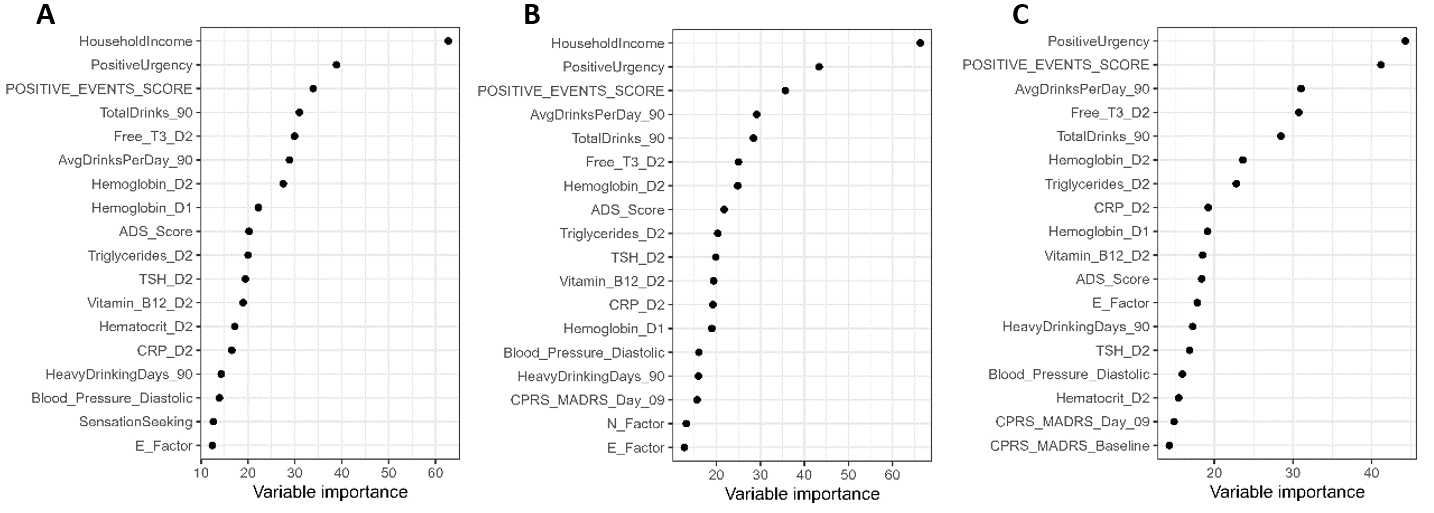


**Supplementary Figure S1**. Random forest variable importance scores for the top 90th percentile important variables under 3 different modeling strategies for household income. **(A)** Variable importance scores from a model treating the nine levels of the household income variable as categorical. **(B)** Variable importance scores from a model including the household income variable used in (A) in addition to a numeric variable using the midpoints of the income ranges. Only the categorical variable is among the top 90th percentile variables shown in the plot. **(C)** Variable importance scores from a model including the numeric household income variable without the categorical household income variable. The numeric household income variable was not among the top 90th percentile important variables and is thus not shown.

**References**

1. Koenig LB, Jacob T, Haber JR. Validity of the Lifetime Drinking History: A Comparison of Retrospective and Prospective Quantity-Frequency Measures. Journal of Studies on Alcohol and Drugs. 2009;70(2):296-303.

2. Sobell LC, Sobell MB. Timeline follow-back: A technique for assessing self-reported alcohol consumption. Measuring alcohol consumption: Psychosocial and biochemical methods: Springer; 1992. p. 41-72.

3. Flannery BA, Volpicelli JR, Pettinati HM. Psychometric Properties of the Penn Alcohol Craving Scale. Alcoholism: Clinical and Experimental Research. 1999;23(8):1289-95.

4. Sullivan JT, Sykora K, Schneiderman J, Naranjo CA, Sellers EM. Assessment of alcohol withdrawal: the revised clinical institute withdrawal assessment for alcohol scale (CIWA-Ar). British journal of addiction. 1989;84(11):1353-7.

5. Babor TF, Higgins‐Biddle JC, Saunders JB, Monteiro MG. AUDIT: the alcohol use disorders identification test: guidelines for use in primary health care. World Health Organization; 2001.

6. McLellan A, Kushner H, Metzger D, Peters R, Smith I, Grissom G, et al. Addiction severity index-(ASI)[database record]. APA PsycTests. 1992;10.

7. Skinner HA, Allen BA. Alcohol Dependence Syndrome: Measurement and Validation.

8. Anton RF. Obsessive–compulsive aspects of craving: development of the Obsessive Compulsive Drinking Scale. Addiction. 2000;95(8s2):211-7.

9. First MB, Spitzer RL, Gibbon M, Williams JBW. Structured Clinical Interview for DSM-IV Axis I Disorders (SCID-I), Clinician Version, User’s Guide. Biometrics Research Department, New York State Psychiatric Institute; 1997.

10. First MB, Williams JBW, Karg RS, Spitzer RL. Structured clinical interview for DSM-5 disorders. Clinician Version (SCID-5-CV). 2015.

11. Heatherton TF, Kozlowski LT, Frecker RC, Fagerstrom K-O. The Fagerström test for nicotine dependence: a revision of the Fagerstrom Tolerance Questionnaire. British journal of addiction. 1991;86(9):1119-27.

12. Asberg M Fau - Montgomery SA, Montgomery Sa Fau - Perris C, Perris C Fau - Schalling D, Schalling D Fau - Sedvall G, Sedvall G. A comprehensive psychopathological rating scale. (0065-1591 (Print)).

13. Wechsler D. Wechsler Abbreviated Scale of Intelligence. PsycTESTS Dataset: American Psychological Association (APA); 1999.

14. Costa PT, McCrae RR. The Revised NEO Personality Inventory (NEO-PI-R). The SAGE Handbook of Personality Theory and Assessment: Volume 2 — Personality Measurement and Testing: SAGE Publications Ltd; 2008. p. 179-98.

15. Bernstein DP, Fink L, Handelsman L, Foote J. Childhood Trauma Questionnaire. PsycTESTS Dataset: American Psychological Association (APA); 1994.

16. McFarlane A, Clark CR, Bryant RA, Williams LM, Niaura R, Paul RH, et al. The impact of early life stress on psychophysiological, personality and behavioral measures in 740 non-clinical subjects. Journal of integrative neuroscience. 2005;4(01):27-40.

17. The Whoqol G. The World Health Organization quality of life assessment (WHOQOL): Development and general psychometric properties. Social Science &amp; Medicine. 1998;46(12):1569-85.

18. Posner K, Brown GK, Stanley B, Brent DA, Yershova KV, Oquendo MA, et al. The Columbia–Suicide Severity Rating Scale: Initial Validity and Internal Consistency Findings From Three Multisite Studies With Adolescents and Adults. American Journal of Psychiatry. 2011;168(12):1266-77.

19. Cohen S, Kamarck T, Mermelstein R. A global measure of perceived stress. Journal of health and social behavior. 1983:385-96.

20. Kessler RC, Adler L, Ames M, Demler O, Faraone S, Hiripi EVA, et al. The World Health Organization adult ADHD self-report scale (ASRS): a short screening scale for use in the general population. Psychological Medicine. 2005;35(2):245-56.

21. Buysse DJ, Reynolds CF, Monk TH, Berman SR, Kupfer DJ. The Pittsburgh sleep quality index: A new instrument for psychiatric practice and research. Psychiatry Research. 1989;28(2):193-213.

22. Buss AH, Perry M. The aggression questionnaire. Journal of personality and social psychology. 1992;63(3):452.

23. Lynam D, Smith GT, Cyders MA, Fischer S, Whiteside SA. The UPPS-P: A multidimensional measure of risk for impulsive behavior. Unpublished technical report. 2007.

24. Patton JH, Stanford MS, Barratt ES. Factor structure of the Barratt impulsiveness scale. Journal of clinical psychology. 1995;51(6):768-74.

25. Gearhardt AN, Corbin WR, Brownell KD. Preliminary validation of the Yale Food Addiction Scale. Appetite. 2009;52(2):430-6.

26. Delgado C, Baweja M, Crews DA-O, Eneanya NA-O, Gadegbeku CA, Inker LA, et al. A Unifying Approach for GFR Estimation: Recommendations of the NKF-ASN Task Force on Reassessing the Inclusion of Race in Diagnosing Kidney Disease. (1533-3450 (Electronic)).

27. Stekhoven DJ. missForest: Nonparametric Missing Value Imputation using Random Forest2022 2022.

28. Breiman L, Cutler A, Liaw A, Wiener M. randomForest: Breiman and Cutlers Random Forests for Classification and Regression. CRAN: Contributed Packages: The R Foundation; 2002.

29. Friedman J, Hastie T, Tibshirani R. Regularization Paths for Generalized Linear Models via Coordinate Descent. Journal of Statistical Software. 2010;33(1).

30. Singal AK, Bataller R, Ahn J, Kamath PS, Shah VH. ACG Clinical Guideline: Alcoholic Liver Disease. Official journal of the American College of Gastroenterology | ACG. 2018;113(2).

31. Skinner HA, Sheu WJ. Reliability of alcohol use indices. The Lifetime Drinking History and the MAST. J Stud Alcohol. 1982;43(11):1157-70.

32. Strong DR, Uebelacker L Fau - Fokas K, Fokas K Fau - Saritelli J, Saritelli J Fau - Matsko S, Matsko S Fau - Abrantes AM, Abrantes Am Fau - Schonbrun Y, Schonbrun Y. Utilization of evidence-based smoking cessation treatments by psychiatric inpatient smokers with depression. (1935-3227 (Electronic)).

33. Spielberger CD. State-trait anxiety inventory for adults. 1983.

34. Norbeck JS. Modification of Life Event Questionnaires for Use with Female Respondents. Research in Nursing & Health. 1984;7(1):61-71.
